# Phylogeny and Evolution of *Cocconeiopsis* (Cocconeidaceae) as Revealed by Complete Chloroplast and Mitochondrial Genomes

**DOI:** 10.3390/ijms25010266

**Published:** 2023-12-23

**Authors:** Feichao Du, Yuhang Li, Kuidong Xu

**Affiliations:** 1 Laboratory of Marine Organism Taxonomy and Phylogeny, Institute of Oceanology, Chinese Academy of Sciences, Qingdao 266071, China; dufc@qdio.ac.cn (F.D.); liyuhang@qdio.ac.cn (Y.L.); 2 Qingdao Key Laboratory of Marine Biodiversity and Conservation, Institute of Oceanology, Chinese Academy of Sciences, Qingdao 266071, China; 3 University of Chinese Academy of Sciences, Beijing 100049, China; 4 Laoshan Laboratory, Qingdao 266237, China

**Keywords:** diatoms, organellar genome, comparative genomics, phylogeny, divergence time

## Abstract

The genus *Cocconeiopsis* was separated from *Navicula*, but its systematic position is in debate. We sequenced the complete chloroplast and mitochondrial genome of Cocconeidaceae for the first time with *Cocconeiopsis kantsiensis* and investigated its phylogeny and evolutionary history. Results showed that the plastid genome was 140,415 bp long with 167 genes. The mitochondrial genome was 43,732 bp long with 66 genes. Comparative analysis showed that the plastid genome structure of *C. kantsiensis* was most similar to those of three *Navicula* species and *Halamphora americana*, and its size was significantly smaller than that of a monoraphid species. Its mitochondrial genome was similar to that of related species except for *Phaeodactylum tricornutum*. The multigene phylogeny reconstruction showed that *Cocconeiopsis* was sister to *Didymosphenia* but distant from Naviculaceae. The two-gene phylogenetic analysis containing 255 species showed *Cocconeiopsis* was sister to *Cocconeis*, and distant from Naviculaceae as well. Divergence time estimation indicates the common ancestor of cocconeid species occurred about 62.8 Ma and *Cocconeiopsis* diverged with monoraphid *Cocconeis* about 58.9 Ma. Our results support the assignment of *Cocconeiopsis* to Cocconeidaceae and that monoraphid cocconeids were likely evolved from the lineage of *Cocconeiopsis*.

## 1. Introduction

The genus *Cocconeiopsis* was established by Witkowski et al. [1] to accommodate ten *Navicula* species similar to *N. orthoneoides* Hustedt. This genus is characterized by a flat discoid to linear-elliptic valve, a filiform and straight raphe internally running on an elevated raphe sternum, external simple and usually expanded raphe endings, terminal ones distant from apices, areolae loculate with external vela, and internal round pores [1]. Subsequently, Riaux–Gobin and Witkowski [2] added two new species, *C. juandenovensis* and *C. discoides*. Up to now, this genus contains 12 species [3].

The systematic position of *Cocconeiopsis* remains unclear. Morphologically, they have two raphes and laterally symmetric valves, suggesting a close relationship with Naviculaceae. However, the presence of elliptical valves, areolae loculate with external vela and internal round pores, and shallow mantles makes it also resemble the genus *Cocconeis*. Initially, Witkowski et al. [1] established and assigned this genus to “Naviculaceae (in traditional sense)”. However, Riaux–Gobin and Witkowski [2] did not confirm this assignment and classified it as Baccilariophyceae *incertae sedis*. In a more recent modification by Cox [4], this genus was moved to Cocconeidaceae. Currently, the molecular phylogenetic study of *Cocconeiopsis* is lacking and its exact phylogenetic position remains unknown.

The chloroplast and mitochondrial genome are now widely used for phylogenomic and molecular evolutionary studies due to their conserved nature and relatively high substitution rate [5,6,7]. Kowallik et al. [8] sequenced the chloroplast of a marine centric diatom, *Odontella sinensis*, providing the first organelle genome of a diatom. Subsequently, the chloroplast and mitochondrial genome of a biraphid diatom, *Phaeodactylum tricornutum*, were sequenced, revealing several novel features, including gene transfer from plastid to host nucleus and a large mitochondrial genome [9,10]. Recently, Górecka et al. [11] provided for the first time the complete plastid and mitochondrial genomes of a monoraphid diatom, *Schizostauron trachyderma*, and resolved the phylogenetic positions of this species based on the concatenated multigene. At present, there are over twenty raphid species whose organelle genomes have been deposited in GenBank, but no one belongs to the cocconeid group.

In this study, we sequenced the plastid and mitochondrial genome of *Cocconeiopsis kantsiensis* and compared the genomic structure and nucleotide composition with those of related species. The phylogenetic position of *C. kantsiensis* was analyzed by using shared organelle protein-coding genes and a two-gene dataset (SSU rDNA–*rbc*L). This study aims to reveal the systematic position of the genus *Cocconeiopsis* and to better understand its evolutionary relationship.

## 2. Results

### 2.1. Morphology

**Figure 1:**
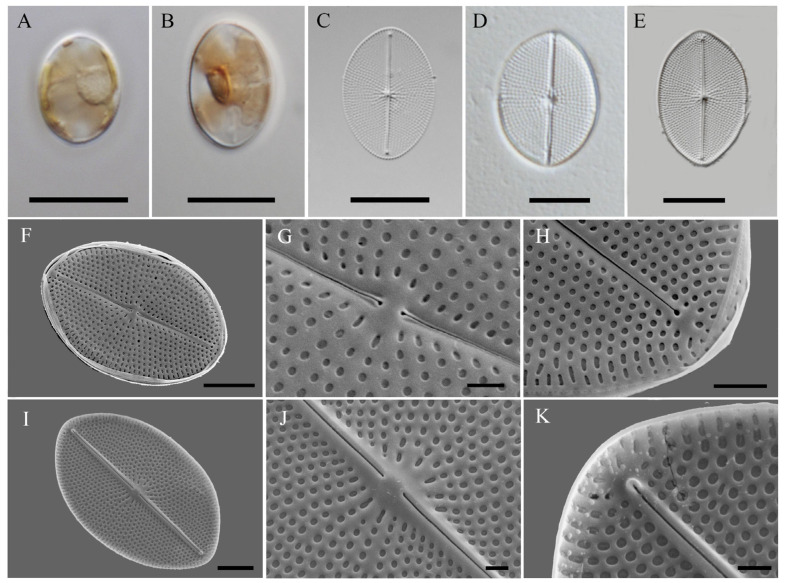
LM and SEM photographs of *Cocconeiopsis kantsiensis*. (**A**,**B**) live cells with plastids in valve view; (**C**–**E**) cleaned frustules in LM; (**F**–**H**) external view of the valve in SEM; (**G**) external central area showing the expanded central raphe endings; (**H**) external distal raphe ending expanded distant from valve margin; (**I**–**K**) internal view of the valve in SEM; (**J**) internal valve central area with simple raphe endings; (**K**) internal raphe fissures terminate in helictoglossae at the apices. Scale bars: 10 μm (**A**–**E**); 5 μm (**F**,**I**); 2 μm (**H**); 1 μm (**G**,**J**,**K**).

Figure 1A–E show LM images of a solitary cell. One plastid with four lobes with a central bridge containing a pyrenoid. The nucleus lies centrally (Figure 1A,B). Valves shallow, broadly elliptical with rounded apices. 19.9–22.6 µm long, 13.2–15.7 µm in width (n = 20). Valve central area small and roundish. The transapical striae are uniseriate, 18–22 in 10 µm, strongly radiate and punctate.

SEM images (Figure 1F–K) show that, externally, raphe central fissures filiform, straight and expanded. Distal raphe endings expanded, rounded, and distant from the valve apices. Internal raphe fissures straight and terminate in small helictoglossae apart from apices. Straight raphe internally running on an elevated raphe sternum. Areolae round on most of the valve surface but elliptic near the margin and central area.

### 2.2. General Characterization of C. kantsiensis Plastid Genome

**Figure 2:**
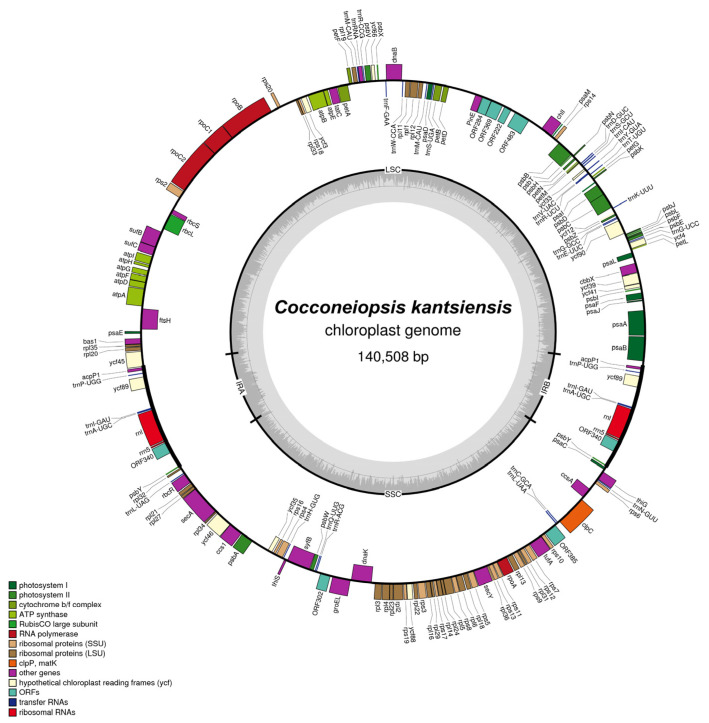
Map of the plastid genome of *C. kantsiensis*. The darker grey in the inner circle represents the GC content. The genes belonging to different functional groups are color-coded.

The plastid genome of *C. kantsiensis* was 140,415 bp in length and presented a typical quadripartite structure with a large single-copy region (LSC) of 76,086 bp, a small single-copy region (SSC) of 44,932 bp and a pair of inverted repeat regions (IR) of 9699 bp each (Figure 2). The GC content of the complete *C. kantsiensis* plastid genome was 31.8%, and the three main regions—LSC, SSC, and IR—were calculated to be about 30.3, 31.2, and 39.1%, respectively. The higher GC content in the IR region of *C. kantsiensis* might result from two rRNA genes which have high GC content [12].

The plastid genome of *C. kantsiensis* contained a total of 167 genes, including 130 protein-coding genes (PCGs), 7 open-reading frames (ORFs), 28 tRNA genes and 2 rRNA genes. None of the genes contained intron. The distribution of PCGs in the plastid genome of *C. kantsiensis* showed that 74, 52, and 4 genes were located in the LSC, SSC, and IR regions, respectively. Among the 7 ORFs, *orf284* encoded a putative integrase/recombinase protein, but its sequence was incomplete. Note that *orf284* was closed to the *PinE* that was inferred to encode a functional protein serine recombinase. Hence, we speculated that *orf284* is likely a pseudogene originating from *PinE* [11].

### 2.3. Comparative Analysis of C. kantsiensis Plastid Genome

The plastid genomes of nine biraphid diatoms and one monoraphid diatom were chosen to compare with that of *C. kantsiensis* (Table 1). The plastid genome size of *C. kantsiensis* (140,415 bp) is similar to those of three *Navicula* species (*N. arenaria*, *N. avium*, and *N. tsukamotoi*) and *Halamphora americana*, ranging from 136,746 to 147,331 bp; it is significantly smaller than that of *Pleurosigma intermedium* (174,382 bp), *Seminavis. robusta* (150,905), and the monoraphid species *Schizostauron trachyderma* (187,029 bp), but is larger than that of *Phaeodactylum tricornutum* (117,369 bp), *Didymosphenia geminata* (117,972), and *Fistulifera saprophila* (122,456 bp). The GC contents of these 11 species are similar (29.6–32.6%). The length of the IR regions of the *C. kantsiensis* (9699 bp) plastid genome is similar to that of other biraphid species except *Phaeodactylum tricornutum* (6912 bp) and *Didymosphenia geminate* (6996 bp), but significantly smaller than that of the monoraphid species *Schizostauron Trachyderma* (17,237 bp). Moreover, monoraphid species (197 genes) have more plastid genes when compared with biraphid species (157–175 genes), which is attributed to a large number of ORFs (42). However, the result was based on only one plastid genome, and further studies involving more plastid genomes of monoraphid diatoms are necessary to confirm this conclusion.

**Figure 3:**
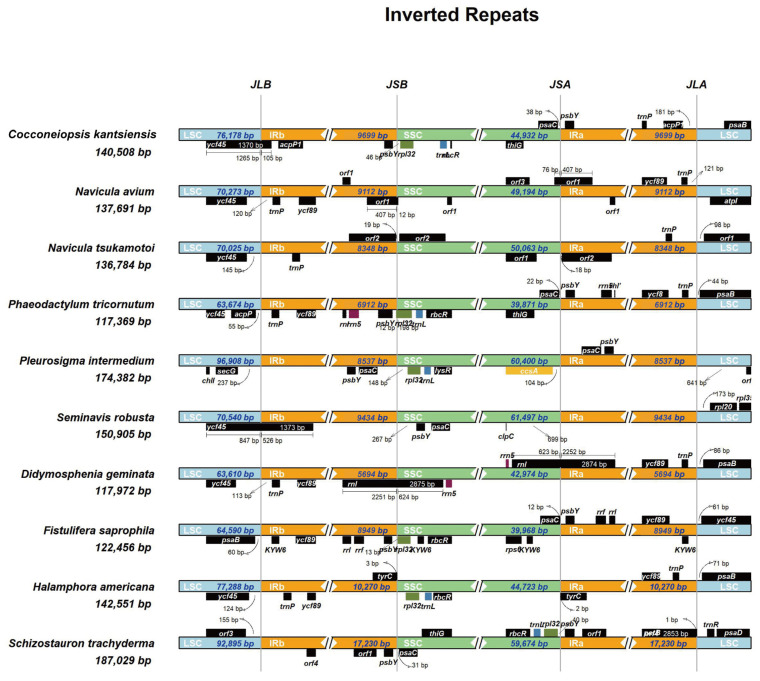
Comparison of the LSC/IR and IR/SSC junction of the plastid genome between *C. kantsiensis* and related species.

Comparative analysis of the boundaries of the junction sites of the *C. kantsiensis* plastid genome with nine species, including eight biraphid species (*Navicula avium*, *N. tsukamotoi*, *Phaeodactylum tricornutum*, *Pleurosigma intermedium*, *Seminavis robusta*, *Didymosphenia geminate*, *Fistulifera saprophila*, and *Halamphora americana*), and one monoraphid species, *Schizostauron trachyderma*, revealed that the IR region expansion was different within diatom species (Figure 3). The JLB (the junction between IRb and LSC) extended 105, 1373 and 3 bp into the pseudogene *ycf45* of *C. kantsiensis*, *Seminavis robusta*, and *Halamphora karadagensis*, respectively. The *rpl32* was located in the SSC and 46, 148, 13, and 122 bp away from the JSB (the junction between IRb and SSC) in *C. kantsiensis*, *Pleurosigma intermedium*, *Fistulifera saprophila*, and *Halamphora americana* plastid genomes, respectively. However, in *Phaeodactylum tricornutum*, the JSB expanded 12 bp into *rpl32*. Notably, in the monoraphid species *Schizostauron trachyderma*, the *rpl32* was close to the JSA (the junction between IRa and SSC), within 40 bp. The *psaC* was also located in the SSC and 38 and 22 bp away from the JSA in *C. kantsiensis* and *Phaeodactylum tricornutum*, respectively. In addition, the *acpP1* (43 bp) was only found in IR regions of *C. kantsiensis* and was 181 bp away from the JLA (the junction between IRa and LSC).

### 2.4. General Characterization of C. kantsiensis Mitochondrial Genome

**Figure 4:**
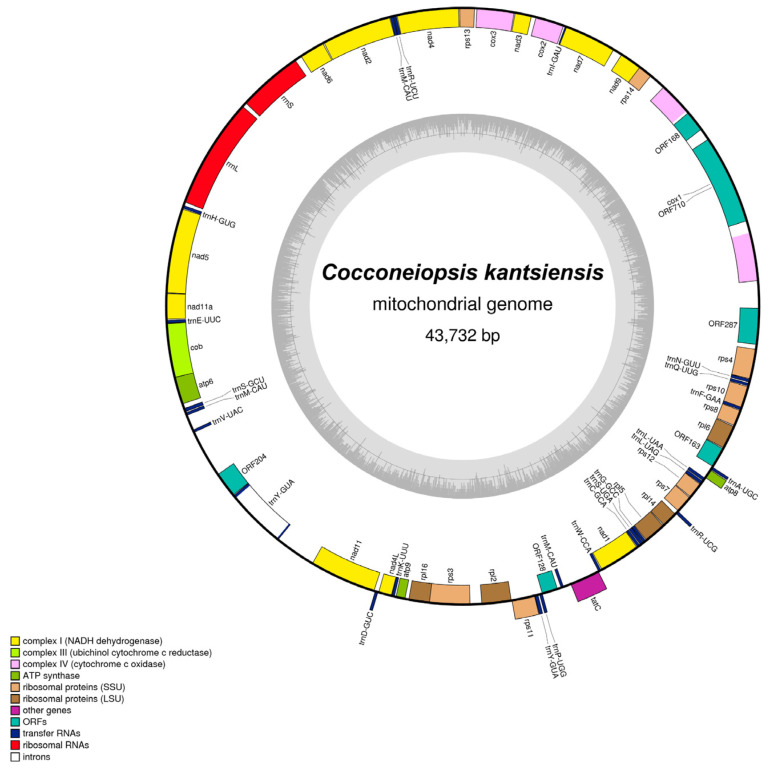
Map of the mitochondrial genome of *C. kantsiensis*. The darker grey in the inner circle represents the GC content. The genes belonging to different functional groups are color-coded.

The complete circular mitochondrial genome of *C. kantsiensis* was 43,732 bp in length and 28.3% in GC content (Figure 4). It encoded a total of 66 genes, including 33 PCGs that included the two conserved subunits of *Nad11* in diatoms, 6 ORFs, 2 rRNA genes, and 25 tRNA genes. In these 33 PCGs, the TTA is the most common start codon (25) and is followed by the CTA (3), ATG (3), ATT (1), and TCA (1) codons. Two introns were found in the *C. kantsiensis* mitochondrial genome: one located in the *trnY–GUG* genes with 1387 bp, the other one being a group II intron of 3270 bp in length which classically interrupts the *cox1* [18], with two intronic *orf* (*orf168* and *orf710*). The *C. kantsiensis* mitochondrial genome has 25 tRNA, which is almost enough to satisfy all translation requirements, but like other heterokonts, *C. kantsiensis* has lost the *tRNA–Thr* [10]. In addition, most PCGs (35/37) are encoded on the same strand, as previously found in *Phaeodactylum tricornutum* and *Asterionella formosa* [10,19].

### 2.5. Comparative Analysis of C. kantsiensis Mitochondrial Genome

The mitochondrial genomes of seven related biraphid species and one monoraphid species were selected to compare with *C. kantsiensis* (Table 2). The size (43,732) and overall GC content (28.30%) of the *C. kantsiensis* mitochondrial genome are similar to those of other related species except for *Phaeodactylum tricornutum*, which has the largest mitochondrial genome size and the highest GC content due to the large repeat regions [10]. The total gene number (66) and composition of the *C. kantsiensis* mitochondrial genome are well within the range of these related species (62–70), but the number is higher than that of the monoraphid species *Schizostauron trachyderma* (59).

**Figure 5:**
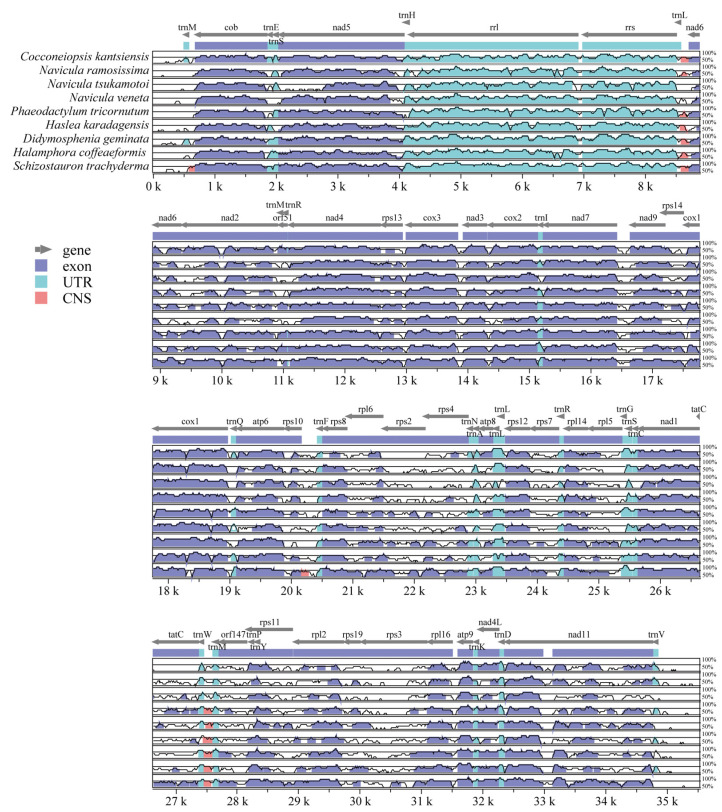
Visualization of the mitochondrial genome alignment between *C. kantsiensis* and related species in mVISTA. The top grey arrows show the position and direction of each gene. The vertical scale represents the identity ranging from 50 to 100%.

mVISTA program analysis was performed to identify the genomic divergences events (Figure 5). Our results showed the tRNA and rRNA genes were relatively conserved in all nine species, and highly divergent regions were detected within protein-coding genes, including *nad2*, *nad11*, *rpl2*, *rpl5*, *rpl6*, *rps2*, *rps3*, *rps4*, *rps7*, *rps10*, *rps14*, *rps19*, *atp8*, and *tatC*, as well as the intergenic non–coding regions such as *trnL*–*nad6*, *trnW*–*trnM*.

### 2.6. Phylogenetic Analysis of C. kantsiensis Plastid and Mitochondrial Genome

**Figure 6:**
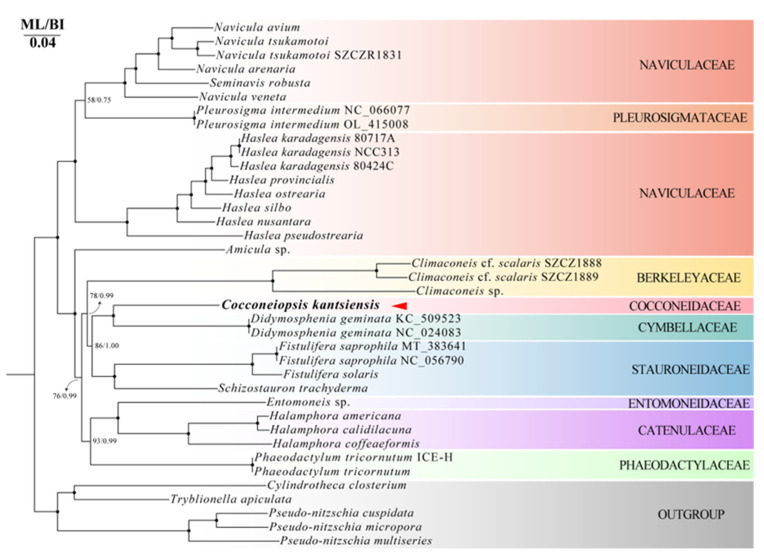
Maximum likelihood (ML) and Bayesian inference (BI) phylogenetic trees based on the concatenated 78 shared protein-coding from 38 plastid genomes of diatoms. The values on each node indicate ML bootstrap and Bayesian posterior probabilities (%), respectively. Only bootstrap values over 50% are shown on the tree. “●” indicates ML/BI = 100/1.00.

**Figure 7:**
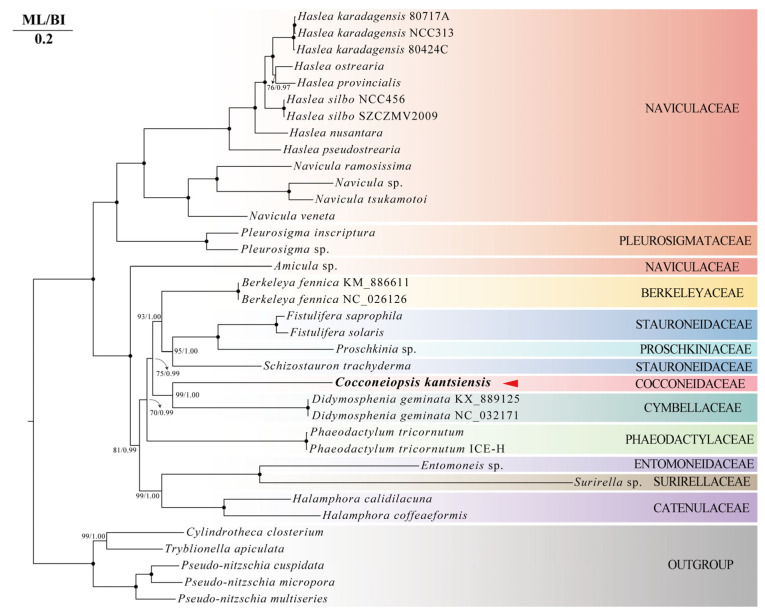
Maximum likelihood (ML) and Bayesian inference (BI) phylogenetic trees based on the concatenated 18 shared protein-codings from 36 mitochondrial genomes of diatoms. The values on each node indicate ML bootstrap and Bayesian posterior probabilities (%), respectively. Only bootstrap values over 50% are shown on the tree. “●” indicates ML/BI = 100/1.00.

We inferred the phylogeny by using 38 pennate diatom plastid genomes, as well as 36 pennate diatom mitochondrial genomes, respectively, with five Bacillariaceae species as outgroups. The ML and BI phylogenetic trees based on plastid genomes (Figure 6) showed that *C. kantsiensis* formed a strongly supported clade together with two *Didymosphenia* species (IQ–TREE ultrafast bootstrap value = 100, Mrbayes posterior probability = 1.00). This clade is sister to the clade comprising *Fistulifera* and *Schizostauron*. While in the ML and BI phylogenetic trees based on mitochondrial genomes (Figure 7), *C. kantsiensis* formed a strongly supported clade together with two *Didymosphenia* species (IQ–TREE ultrafast bootstrap value = 99, Mrbayes posterior probability = 1.00). This clade is sister to the clade comprising *Fistulifera*, *Berkeleya*, *Proschkinia*, and *Schizostauron*. Moreover, in all molecular phylogenetic trees, *C. kantsiensis* has a distant relationship with Naviculaceae.

### 2.7. Phylogenetic Analysis and Divergence Time Estimation Based on a Two-Gene Dataset

**Figure 8:**
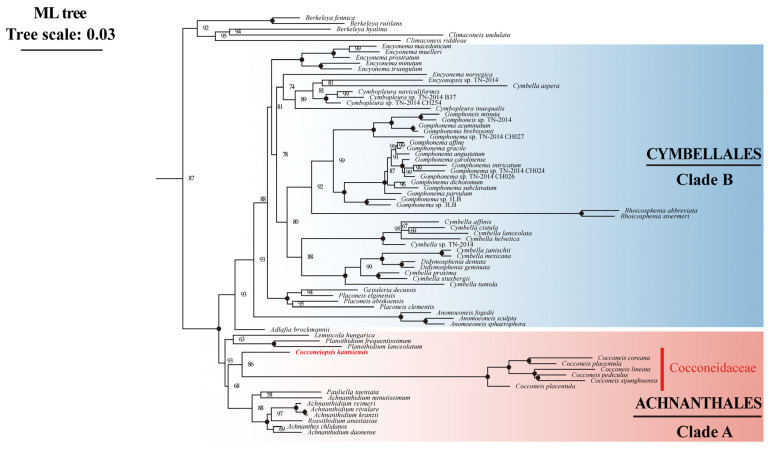
The part of the maximum likelihood (ML) phylogenetic tree based on the concatenated two-gene dataset (SSU rDNA–*rbc*L) from 255 diatoms. The values on each node indicate ML bootstrap (%). Only bootstrap values over 50% are shown on the tree. “●” indicates ML = 100. (See Figure S1 for the complete phylogenetic tree.)

Phylogenetic analysis based on a two-gene dataset (SSU rDNA–*rbc*L) containing 255 species was performed. ML and BI phylogenetic results both showed that *Cocconeiopsis* was placed in Cocconeidaceae and was sister to *Cocconeis* (IQ–TREE ultrafast bootstrap value = 86, Mrbayes posterior probability = 0.77), but distant from the Naviculaceae (Figure 8, Figures S1 and S2). Cocconeidaceae was in a larger clade (Clade A) with Achnanthidiaceae (Figure 8). Moreover, both of the phylogenetic analysis based on two-gene and organelle PCGs support the close phylogenetic relationship between Achnanthales (Clade A) and Cymbellales (Clade B), which is consistent with previous studies based on single-gene SSU rDNA and a three-gene (SSU rDNA–*rbc*L-*psb*C) dataset [23,24].

**Figure 9:**
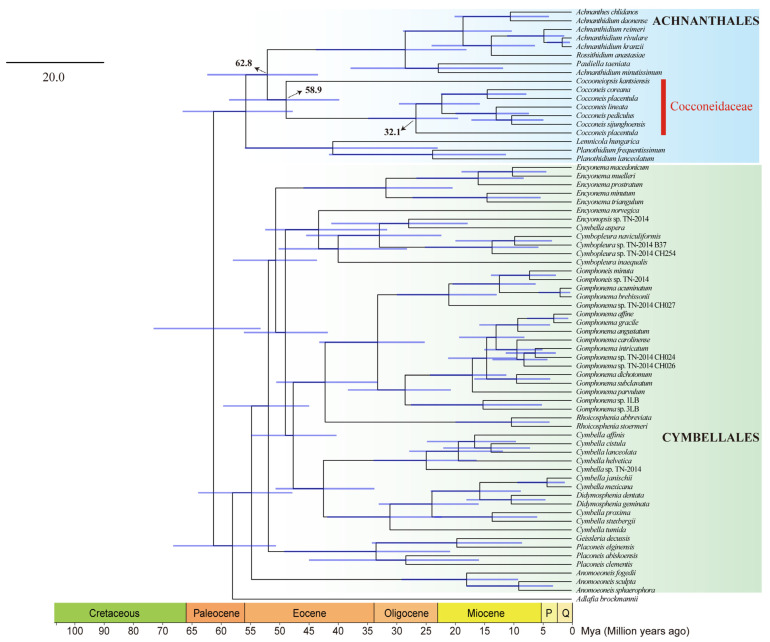
The part of the time-calibrated divergence time estimation of 255 diatoms based on a two-gene dataset (SSU rDNA–*rbc*L) within the ML framework. The red nodes represent the calibration point and blue bars represent the 95% highest posterior density (HPD). (See Figure S3 for the complete divergence time estimate.)

Divergence time estimation calibrated by five fossil records within an ML framework (Figure 9 and Figure S3) displayed that the origin of raphid pennates was in the Cretaceous period (101.6 Ma, 95% HPD: 92.6–114.4 Ma). Cocconeidaceae and Achnanthidiaceae were estimated to have diverged from their common ancestor about 62.8 Ma (95% HPD: 52.3–75.2 Ma). Furthermore, the divergence time between biraphid cocconeid *Cocconeiopsis* and the common ancestor of monoraphid cocconeids was inferred to have occurred about 58.9 Ma (95% HPD: 48.0–70.7 Ma). The origin of *Cocconeis* occurred about 32.1 Ma (95% HPD: 23.4–42.0 Ma).

## 3. Discussion

The plastid genomes of *C. kantsiensis* presented a typical quadripartite structure and a size more similar to those of several biraphid species than those of monoraphid species. There were no genes containing intron in the *C. kantsiensis* plastid genome, which is consistent with all reported diatom plastid genomes except that of *Seminavis robusta* [14]. In addition, previous studies showed that plastid gene loss frequently occurred in diatoms [15]; likewise, the *petJ* and *tsf* genes were also lost in the *C. kantsiensis* plastid genome (Figure 2). It is now clear that *petJ* was transferred to the nucleus in the early evolution in diatoms [15]. The *tsf* is a translation factor gene, which is also missing in other naviculoid species [13], while in *Phaeodactylum tricornutum*, this gene is presented in both the nuclear and plastid genomes, indicating an ongoing process of endosymbiotic gene transfer [9]. Moreover, the *bas1*, *syfB*, *thiG*, and *thiS* were annotated in the *C. kantsiensis* plastid genome, and are often absent or present as pseudogenes in other diatoms [25].

IR region expansion and contraction is the main reason for the variation in the sizes of plastid genomes and contributes to gene duplications in diatoms [26,27]. In this study, the IR regions of ten biraphid diatoms plastid genomes ranged from 6912 to 10,269 bp (Table 1), indicating they are relatively conserved in this group. However, a recent study has reported that a naviculoid species, *Climaconeis* cf. *scalaris*, has a huge IR region up to 78,313 bp due to an extreme IR expansion event [28]. Hence, more plastid genomes of naviculoid species should be sequenced, thus providing us more complete information to investigate the IR regions variation. In addition, the presence of *acpP1* in each of the two IR regions suggests that an ancient duplication event occurred in the ancestor of *C. kantsiensis* because the *acpP* was considered to undergo multiple independent duplications in the *acpP1/2*-containing plastid genomes [27].

The multigene phylogenetic analysis based on plastid and mitochondrial genomes confirms the distant evolutionary relationship between *Cocconeiopsis* and Naviculaceae, which is different from the high similarity in the size and GC content of plastid and mitochondrial genomes between *C. kantsiensis* and *Navicula* species (Table 1 and Table 2). The two-gene phylogenetic analysis further showed that *Cocconeiopsis* was sister to *Cocconeis*, and that these cocconeids were placed in a larger clade with other Achnanthidiaceae species. These results support the establishment of the genus *Cocconeiopsis* separated from Naviculaceae, and assigning this genus to Cocconeidaceae [1,4]. Furthermore, divergence time estimation indicates the common ancestor of cocconeid species occurred about 62.8 Ma. The mass extinction event that occurred in this period caused the loss of about 85% of species on earth, which may have offered many niches for the emergence of these cocconeid species [29]. Cocconeid species could be divided into two groups according to the valve morphology and the number of raphes. For example, *Cocconeis* is heterovalvy: the concave valve is accompanied by a raphe system and the convex valve is not [30]; in *Cocconeiopsis*, the valves are flat and each has a raphe system. Previous studies argued that monoraphid diatoms were evolved from biraphid species [30,31,32]. In this study, *Cocconeiopsis* was in the basal position of the clade Cocconeidaceae and occurred about 58.9 Ma, earlier than the origin time of *Cocconeis*, about 32.1 Ma, indicating that these monoraphid cocconeid groups were likely derived from the biraphid cocconeid group *Cocconeiopsis*.

## 4. Materials and Methods

### 4.1. Sampling, Cultivation, and Morphological Observation

Surface muddy sediments with benthic diatoms were sucked up by using a glass tube from an intertidal sand beach in the Huiquan Bay, Qingdao City. The diatom cells were isolated using capillary pipettes and cultivated in 250 mL cell culture flasks with 100 mL F/2 medium. Cultures were maintained at 20–22 °C under a low light intensity (25–30 µmol photo/m^2^/s) with a light/dark cycle of 12:12 h. Five millilitres of diatom culture were fixed with 2.5% glutaraldehyde and then cleaned with hydrogen peroxide to remove organic components of frustules [33].

Morphological observation followed the method described in our previous study [34]: for light microscopy (LM) observation, cleaned samples were pipetted onto the coverslips, air-dried, and mounted on glass slides with Mountmedia (Wako Pure Chemical Industries, Ltd., Osaka, Japan). LM microphotographs of cleaned frustules were taken by using a Zeiss Imager Z2 microscope (Carl Zeiss Microimaging GmbH, Jena, Germany) with differential interference contrast (DIC). For scanning electron microscopy (SEM) observation, cleaned frustules were placed on round coverslips, air-dried, and coated with osmium. SEM observation was performed by using a Hitachi S–4800 (Hitachi, Ltd., Tokyo, Japan).

### 4.2. DNA Extraction and Sequencing

The cells of *C. kantsiensis* were harvested at the exponential growth phase by centrifuging 50 mL cultures at 5000× *g* for 5 min. The sample was quickly frozen in liquid nitrogen and stored at −80 °C for DNA extraction. Total DNA was extracted using the Plant Genomic DNA Kit (Tiangen Biotech Co., Beijing, China) according to the manufacturer’s instructions. Half of the total DNA was sent to Beijing BerryGenomics Biotechnology Co., Ltd. (Beijing, China) for library construction and Illumina sequencing. The rest of the total DNA was used for small-subunit ribosomal DNA (SSU rDNA) and chloroplast-encoded gene *rbc*L amplification by polymerase chain reaction (PCR). Forward and reverse strands were amplified using primers (Table 3). The PCR cycles of the two markers follow [35]. The PCR products were purified by using a TIANgel Midi Purification Kit (Tiangen Biotech Co., Beijing, China) and sequenced by Tsingke Biotechnology Co., Ltd. (Beijing, China). These sequences were deposited in the GenBank (SSU rDNA OR712156 and *rbc*L OR700023).

### 4.3. Organelle Genome Assembly and Annotation

Raw data were trimmed by removing adaptors and low-quality reads using Trimmomatic—0.39 (trimwindows = 5; minlength = 25) [39]. De novo assembly was performed using SPAdes v. 3.9.0 [40] with default settings. The annotation of the plastid genome was performing by the GeSeq [41], using the *Navicula veneta* (GenBank: MT383645) plastid genome as a reference. The structural and functional annotation of the mitochondrial genome was performed by online software MITOS (http://mitos.bioinf.uni-leipzig.de/ accessed on 2 June 2023). Protein-coding genes (PCGs) and rRNA genes were annotated by aligning with the mitochondrial genome of *Navicula veneta* (GenBank: MT383644). Apollo software [42] was used to manually correct the boundaries of positions of start and stop codons to ensure the accuracy of the annotation results. A map of the organelle genome was produced by OGDRAW [43]. The annotated complete plastid and mitochondrial genome of *C. kantsiensis* were deposited in GenBank with accession numbers OR699085 and OR699086, respectively.

### 4.4. Phylogenetic Analysis and Divergence Time Estimation

For multigene phylogenies, plastid and mitochondrial protein-coding genes were extracted separately and aligned with related species gene sequences obtained from GenBank by using PhyloSuite [44]. Five diatoms including *Cylindrotheca Closterium*, *Tryblionella apiculate*, *Pseudo–nitzschia cuspidate*, *Ps. micropora* and *Ps. multiseries* served as outgroups. The information of the selected species was listed in Table S1. The number of shared protein-coding genes in selected species is 78 for the plastid genome and 18 for the mitochondrial genome. These sequences were aligned using MAFFT v.7.313 [45] with normal mode. The trimAl was used to trim the alignment with parameter automated1 [46]. These sequences were then concatenated by PhyloSuite [44]. The final size of the alignments was 63,182 bp for the plastid genes alignment and 14,592 bp for the mitochondrial genes alignment. All genes were partitioned by codon position. PartitionFinder 2 was used to select best-fit models for ML and BI analysis [47], according to the Bayesian information criterion (BIC). IQ–TREE v.1.6.8 [48] and Mrbayes v.3.2.7 [49] were conducted to perform the maximum likelihood (ML) and Bayesian inference (BI) for the two alignments, respectively. The ML analysis with 1000 bootstrap was executed with the default settings. The BI program was run for 10^7^ generations with samples every 1000 generations, and the first 25% of trees were discarded as burn-in. Convergence was judged based on the average standard deviation of split frequencies (all less than 0.01) and the ESS values (more than 3000) were analyzed in the R package RWTY [50]. The consensus topology and posterior probabilities of all branches were derived from the remaining trees using a majority-rule consensus approach.

To further infer the phylogenetic position of *C. kantsiensis*, we used a large two-gene dataset (SSU rDNA–*rbc*L) containing 255 species. The information of these genes is listed in Table S2. The phylogenetic analysis based on this two-gene dataset was similar to those of the multigene phylogenies mentioned above and a previous phylogeny study [24]. The final concatenated alignment included 1777 positions, of which 729 columns were SSU rDNA and 1048 were *rbc*L. The *rbc*L gene was partitioned by codon position to select best-fit models for ML and BI analysis. Based on the two-gene dataset (SSU rDNA–*rbc*L), we used the ML tress as the framework to estimate the divergence time of the 255 species by using MCMCTree in PAML v4. 9 [51]. The substitution rate was calculated using baseml first to get the rgene_gamma. We then selected the GTR + G substitution model with an independent-rate clock model to calculate branch lengths, gradient, and Hessian. Five calibration points [52,53,54] were used in this analysis (Table S3). 200,000 samples were discarded as a burn-in, the sample frequency was 10 and the number of samples was 20,000. Finally, FigTree v.1.4.4 and Adobe Illustrator were used to display and edit trees.

## 5. Conclusions

We sequenced and analyzed the plastid and mitochondrial genomes of a biraphid Cocconeidaceae species, *Cocconeiopsis kantsiensis*, for the first time. Comparative analysis showed that the plastid genome structure of *C. kantsiensis* was similar to those of three *Navicula* species (*N. arenaria*, *N. avium*, and *N. tsukamotoi*) as well as that of *Halamphora americana*, but its size was significantly smaller than those of monoraphid species *Schizostauron trachyderma*. the mitochondrial genome was similar to that of other related species except for *Phaeodactylum tricornutum*. Phylogenetic trees constructed based on shared protein-coding genes of plastid and mitochondrial genomes indicate that *Cocconeiopsis* is sister to *Didymosphenia* and has a distant relationship with Naviculaceae. To obtain a more clear phylogenetic position and evolutionary history of *C. kantsiensis*, we performed the phylogenetic analysis based on a two-gene dataset (SSU rDNA–*rbc*L) containing 255 species. The results also suggested that *Cocconeiopsis* was sister to *Cocconeis*, but far from the Naviculaceae, supporting the validity of the generic establishment [1,4]. Moreover, divergence time estimation indicates that the common ancestor of cocconeid species occurred about 62.8 Ma and that *Cocconeiopsis* occurred about 58.9 Ma, earlier than the origin time of *Cocconeis*, indicating that these monoraphid cocconeid groups, including *cocconeis*, were likely derived from the biraphid cocconeid group *Cocconeiopsis*.

## Figures and Tables

**Table 1:** Plastid genome of *Cocconeiopsis kantsiensis* with related species.

Species	*Cocconeiopsis kantsiensis*	*Navicula arenaria*	*Navicula avium*	*Navicula tsukamotoi*	*Phaeodactylum tricornutum*	*Pleurosigma intermedium*	*Seminavis. robusta*	*Didymosphenia geminata*	*Fistulifera saprophila*	*Halamphora americana*	*Schizostauron trachyderma*
Sizes	140,415	147,331	137,691	136,746	117,369	174,382	150,905	117,972	122,456	142,551	187,029
LSC	76,086	80,134	70,273	70,012	63,674	96,638	70,540	63,610	64,590	77,289	92,894
SSC	44,932	47,699	49,194	50,048	39,871	60,400	61,497	40,370	39,968	44,724	59,661
IR	9699	9749	9112	8343	6912	8672	9434	6996	8949	10,269	17,237
GC content	31.80%	30.70%	31.30%	31.40%	32.60%	29.60%	30.92%	31.60%	32.44%	32.00%	30.77%
Total genes	167	165	164	164	162	157	175	160	160	172	197
Reference	This study	[13]	[13]	[13]	[9]	[13]	[14]	[15]	[16]	[17]	[11]

**Table 2:** Mitochondrial genome of *Cocconeiopsis kantsiensis* with related species.

Species	*Cocconeiopsis kantsiensis*	*Navicula ramosissima*	*Navicula tsukamotoi*	*Navicula veneta*	*Phaeodactylum tricornutum*	*Haslea karadagensis*	*Didymosphenia geminata*	*Halamphora coffeaeformis*	*Schizostauron trachyderma*
Sizes	43,732	48,652	50,972	38,897	77,356	40,924	37,765	44,653	41,957
GC content	28.30%	31.11%	28.60%	25.09%	35.01%	29.76%	26.93%	32.90%	27.83%
PCG	33	35	36	35	34	34	35	38	35
tRNA	25	23	25	23	24	22	25	22	22
rRNA	2	3	2	2	2	2	2	2	2
Total genes	66	68	70	62	60	65	62	62	59
Reference	This study	[20]	[21]	[16]	[10]	Accession: OK729586	[22]	[18]	[11]

**Table 3:** Primers were used to amplify SSU rDNA and *rbc*L fragments from *C. kantsiensis*.

Name	Marker	Sequence (5′ to 3′)	Reference
*SSU*1	SSU	AACCTGGTTGATCCTGCCAGT	[36]
ITS1DR	SSU	CCTTGTTACGACTTCACCTTCC	[37]
*rbc*L 66+	*rbc*L	TTAAGGAGAAATAAATGTCTCAATCTG	[35]
*rbc*L 1444−	*rbc*L	GCGAAATCAGCTGTATCTGTW G	[38]

## Data Availability

Molecular data have all been deposited to GenBank with the following link: https://www.ncbi.nlm.nih.gov/genbank/ (accessed on 1 December 2023).

## Supplementary Materials

The following supporting information can be downloaded at: https://www.mdpi.com/article/10.3390/ijms25010266/s1.
