# Supplementary material for: Phylogeny and Evolution of Cocconeiopsis (Cocconeidaceae) as Revealed by Complete Chloroplast and Mitochondrial Genomes
Source: Int J Mol Sci. 2023 Dec 23;25(1):266. doi: 10.3390/ijms25010266 (PMC10778710; doi:10.3390/ijms25010266)
Supplement: Supplementary file 1 [file ijms-25-00266-s001.zip › Figure S1.pdf]

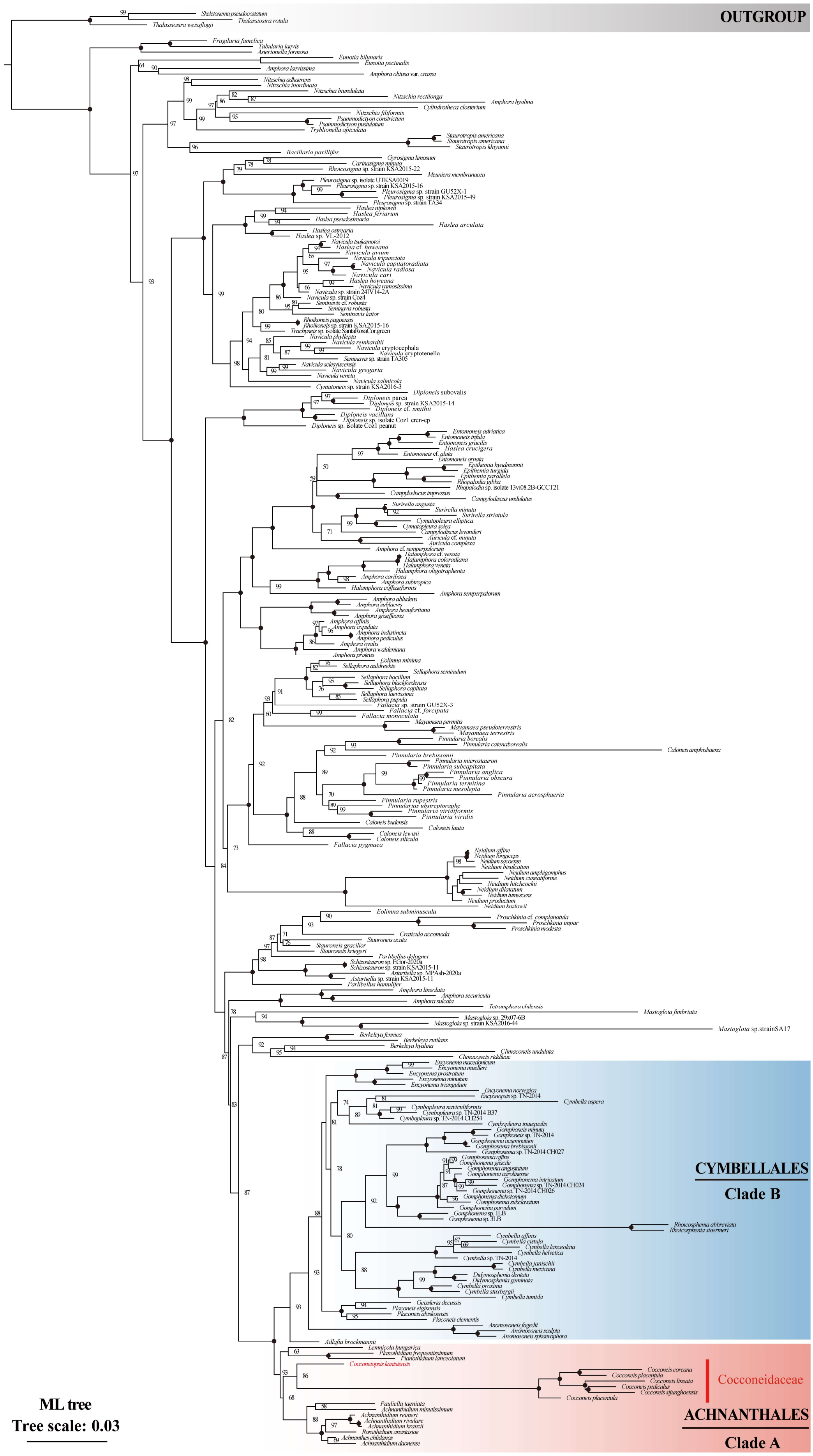

OUTGROUP
Skeletonema pseudocostatum
Thalassiosira rotula
Thalassiosira weissflogii
Fragilaria famelica
Tabularia laevis
Asterionella formosa
Eunotia bilunaris
Eunotia pectinalis
Amphora laevissima
Amphora obtusa var. crassa
Nitzschia adhaerens
Nitzschia inordinata
Nitzschia biundulata
Nitzschia rectilonga
Amphora hyalina
Cylindrotheca closterium
Nitzschia filiformis
Psammodictyon constrictum
Psammodictyon pustulatum
Tryblionella apiculata
Staurotropis americana
Staurotropis khiyamii
Bacillaria paxillifer
Gyrosigma limosum
Carinasigma minuta
Rhoicosigma sp. strain KSA2015-22
Meuniera membranacea
Pleurosigma sp. isolate UTKSA0019
Pleurosigma sp. strain KSA2015-16
Pleurosigma sp. strain GU52X-1
Pleurosigma sp. strain KSA2015-49
Pleurosigma sp. strain TA34
Haslea nipkowii
Haslea feriarum
Haslea pseudostrearia
Haslea arculata
Haslea ostrearia
Haslea sp. VL-2012
Navicula tsukamotoi
Haslea cf. howeana
Navicula avium
Navicula tripunctata
Navicula capitatoradiata
Navicula radiosa
Navicula cari
Haslea howeana
Navicula ramosissima
Navicula sp. strain 24IV14-2A
Navicula sp. strain Coz4
Seminavis cf. robusta
Seminavis robusta
Seminavis latior
Rhoikoneis pagoensis
Rhoikoneis sp. strain KSA2015-16
Trachyneis sp. isolate SantaRosaCor.green
Navicula phyllepta
Navicula reinhardtii
Navicula cryptocephala
Navicula cryptotenella
Seminavis sp. strain TA305
Navicula sclesvicensis
Navicula gregaria
Navicula veneta
Navicula salinicola
Cymatoneis sp. strain KSA2016-3
Diploneis subovalis
Diploneis parca
Diploneis sp. strain KSA2015-14
Diploneis cf. smithii
Diploneis vacillans
Diploneis sp. isolate Coz1 cren-cp
Diploneis sp. isolate Coz1 peanut
Entomoneis adriatica
Entomoneis infula
Entomoneis gracilis
Haslea crucigera
Entomoneis cf. alata
Entomoneis ornata
Epithemia hyndmannii
Epithemia turgida
Epithemia parallela
Rhopalodia gibba
Rhopalodia sp. isolate 13vi08.2B-GCCT21
Campylodiscus impressus
Campylodiscus undulatus
Surirella angusta
Surirella minuta
Surirella striatula
Cymatopleura elliptica
Cymatopleura solea
Campylodiscus levanderi
Auricula cf. minuta
Auricula complexa
Amphora cf. semperpalorum
Halamphora cf. veneta
Halamphora coloradiana
Halamphora veneta
Halamphora oligotraphenta
Amphora caribaea
Amphora subtropica
Halamphora coffeaeformis
Amphora semperpalorum
Amphora abludens
Amphora sublaevis
Amphora beaufortiana
Amphora graeffeana
Amphora affinis
Amphora copulata
Amphora indistincta
Amphora pediculus
Amphora ovalis
Amphora waldeniana
Amphora proteus
Eolimna minima
Sellaphora auldreekie
Sellaphora seminulum
Sellaphora bacillum
Sellaphora blackfordensis
Sellaphora capitata
Sellaphora laevissima
Sellaphora pupula
Fallacia sp. strain GU52X-3
Fallacia cf. forcipata
Fallacia monoculata
Mayamaea permitis
Mayamaea pseudoterrestris
Mayamaea terrestris
Pinnularia borealis
Pinnularia catenaborealis
Caloneis amphisbaena
Pinnularia brebissonii
Pinnularia microstauron
Pinnularia subcapitata
Pinnularia anglica
Pinnularia obscura
Pinnularia termitina
Pinnularia mesolepta
Pinnularia acrosphaeria
Pinnularia rupestris
Pinnularia substreptoraphe
Pinnularia viridiformis
Pinnularia viridis
Caloneis budensis
Caloneis lauta
Caloneis lewisii
Caloneis silicula
Fallacia pygmaea
Neidium affine
Neidium longiceps
Neidium sacoense
Neidium bisulcatum
Neidium amphigomphus
Neidium cuneatiforme
Neidium hitchcockii
Neidium dilatatum
Neidium tumescens
Neidium productum
Neidium kozlowii
Eolimna subminuscula
Proschkinia cf. complanatula
Proschkinia impar
Proschkinia modesta
Craticula accomoda
Stauroneis acuta
Stauroneis gracilior
Stauroneis kriegeri
Parlibellus delognei
Schizostauron sp. EGor-2020a
Schizostauron sp. strain KSA2015-11
Astartiella sp. MPAsh-2020a
Astartiella sp. strain KSA2015-11
Parlibellus hamulifer
Amphora lineolata
Amphora securicula
Amphora sulcata
Tetramphora chilensis
Mastogloia fimbriata
Mastogloia sp. 29x07-6B
Mastogloia sp. strain KSA2016-44
Mastogloia sp.strainSA17
Berkeleya fennica
Berkeleya rutilans
Berkeleya hyalina
Climaconeis undulata
Climaconeis riddleae
Encyonema macedonicum
Encyonema muelleri
Encyonema prostratum
Encyonema minutum
Encyonema triangulum
Encyonema norvegica
Encyonopsis sp. TN-2014
Cymbella aspera
Cymbopleura naviculiformis
Cymbopleura sp. TN-2014 B37
Cymbopleura sp. TN-2014 CH254
Cymbopleura inaequalis
Gomphoneis minuta
Gomphoneis sp. TN-2014
Gomphonema acuminatum
Gomphonema brebissonii
Gomphonema sp. TN-2014 CH027
Gomphonema affine
Gomphonema gracile
Gomphonema angustatum
Gomphonema carolinense
Gomphonema intricatum
Gomphonema sp. TN-2014 CH024
Gomphonema sp. TN-2014 CH026
Gomphonema dichotomum
Gomphonema subclavatum
Gomphonema parvulum
Gomphonema sp. 1LB
Gomphonema sp. 3LB
Rhoicosphenia abbreviata
Rhoicosphenia stoermeri
Cymbella affinis
Cymbella cistula
Cymbella lanceolata
Cymbella helvetica
Cymbella sp. TN-2014
Cymbella janischii
Cymbella mexicana
Didymosphenia dentata
Didymosphenia geminata
Cymbella proxima
Cymbella stuxbergii
Cymbella tumida
Geissleria decussis
Placoneis elginensis
Placoneis abiskoensis
Placoneis clementis
Anomoeoneis fogedii
Anomoeoneis sculpta
Anomoeoneis sphaerophora
CYMBELLALES
Clade B
Adlafia brockmannii
Lemnicola hungarica
Planothidium frequentissimum
Planothidium lanceolatum
Cocconeiopsis kantsiensis
Cocconeis coreana
Cocconeis placentula
Cocconeis lineata
Cocconeis pediculus
Cocconeis sijunghoensis
Cocconeis placentula
Cocconeidaceae
Pauliella taeniata
Achnanthidium minutissimum
Achnanthidium reimeri
Achnanthidium rivulare
Achnanthidium kranzii
Rossithidium anastasiae
Achnanthes childanos
Achnanthidium daonense
ACHNANTHALES
Clade A
ML tree
Tree scale: 0.03

Figure S1. Maximum likelihood (ML) phylogenetic trees based on the concatenated two-gene da-taset (SSU rDNA–*rbc*L) from 255 diatoms. The values on each node indicate ML bootstrap (%). Only bootstrap values over 50% are shown on the tree. "●" indicates ML = 100.
