# Supplementary material for: Phylogeny and Evolution of Cocconeiopsis (Cocconeidaceae) as Revealed by Complete Chloroplast and Mitochondrial Genomes
Source: Int J Mol Sci. 2023 Dec 23;25(1):266. doi: 10.3390/ijms25010266 (PMC10778710; doi:10.3390/ijms25010266)
Supplement: Supplementary file 1 [file ijms-25-00266-s001.zip › Figure S2.pdf]

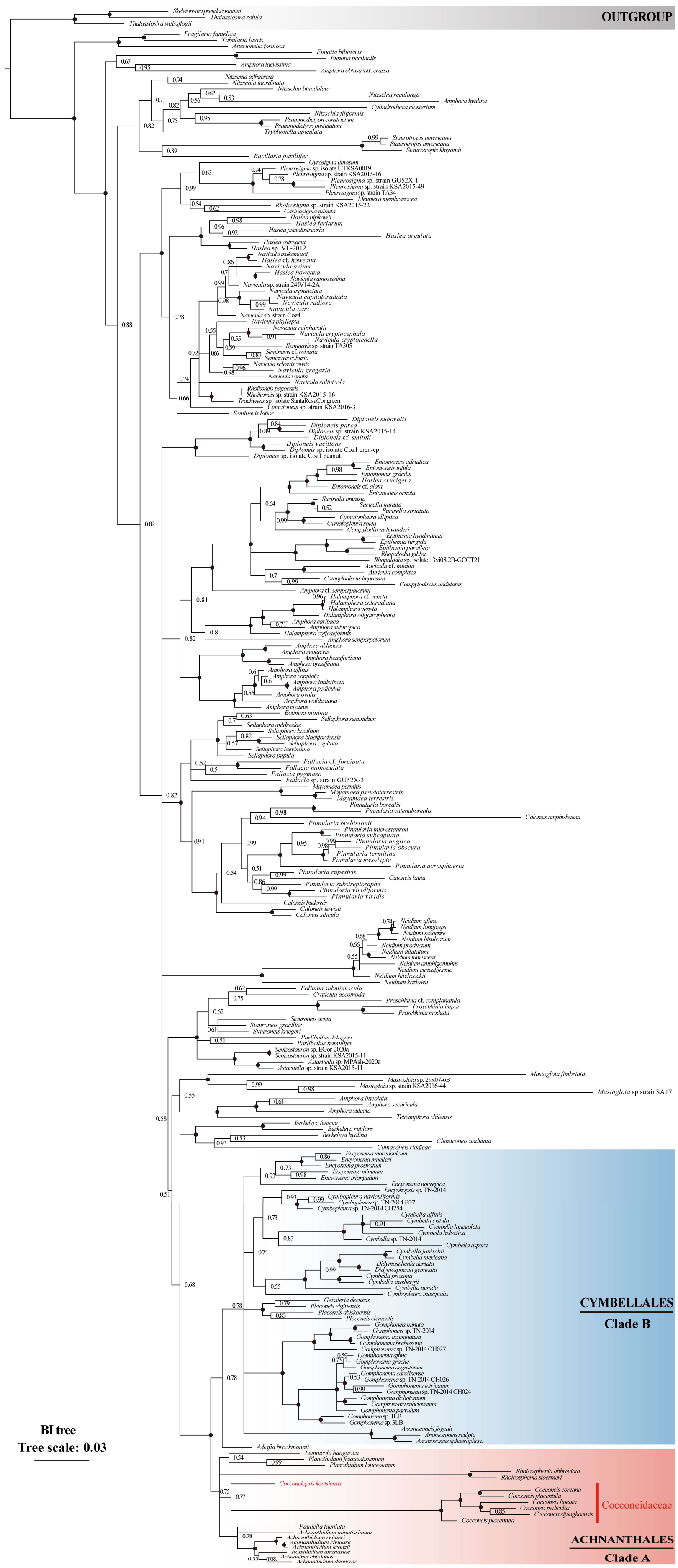
OUTGROUP
CYMBELLALES
Clade B
Cocconeidaceae
ACHNANTHALES
Clade A
BI tree
Tree scale: 0.03

Figure S2. Bayesian inference (BI) phylogenetic trees based on the concatenated two-gene dataset (SSU rDNA–*rbc*L) from 255 diatoms. The values on each node indicate Bayesian posterior proba-bilities. Only bootstrap values over 50% are shown on the tree. "●" indicates BI = 1.00.
