# Supplementary material for: Phylogeny and Evolution of Cocconeiopsis (Cocconeidaceae) as Revealed by Complete Chloroplast and Mitochondrial Genomes
Source: Int J Mol Sci. 2023 Dec 23;25(1):266. doi: 10.3390/ijms25010266 (PMC10778710; doi:10.3390/ijms25010266)
Supplement: Supplementary file 1 [file ijms-25-00266-s001.zip › Figure S3.pdf]

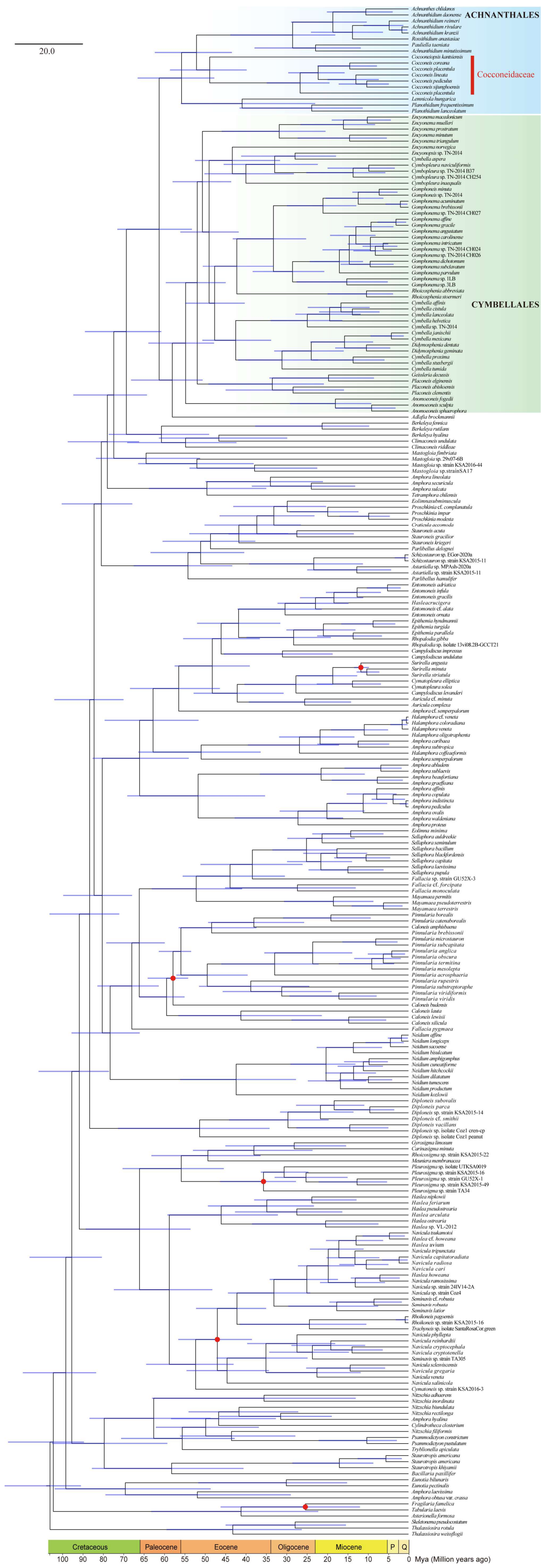
ACHNANTHALES
Cocconeidaceae
CYMBELLALES
20.0
Cretaceous
Paleocene
Eocene
Oligocene
Miocene
P
Q
100 90 80 70 65 60 55 50 45 40 35 30 25 20 15 10 5 0
Mya (Million years ago)

Figure S3. Time-calibrated divergence time estimate of 255 diatoms based on a two-gene dataset (SSU rDNA–*rbc*L) within the ML framework. The red nodes represent the calibration point and blue bars represent the 95% highest posterior density (HPD).
