# Supplementary material for: Phylogeny and Evolution of Cocconeiopsis (Cocconeidaceae) as Revealed by Complete Chloroplast and Mitochondrial Genomes
Source: Int J Mol Sci. 2023 Dec 23;25(1):266. doi: 10.3390/ijms25010266 (PMC10778710; doi:10.3390/ijms25010266)
Supplement: Supplementary file 1 [file ijms-25-00266-s001.zip › Table S3.pdf]

Table S3. Calibration nodes used in this study.

Calibration node	Age (Ma)	References
FA of Surirellaceae	11.6–16.0	[52]
FA of *Fragilaria*	37.7	[53]
FA of *Pleurosigma*	38.0	[53]
FA of *Navicula*	44.0	[53]
FA of *Pinnularia*	64.9-76.8	[54]

Note: FA = the first appearance.
